# Clinical values of neutrophil-to-lymphocyte ratio, platelet-to-lymphocyte ratio, red blood cell distribution width and mean platelet volume in neonatal pulmonary infections

**DOI:** 10.12669/pjms.41.12.12297

**Published:** 2025-12

**Authors:** Juan Du, Qinglin Shi

**Affiliations:** 1 Juan Du, Department Of pediatrics, Mianzhu Peopl’s Hospital, 618200, China; 2 Qinglin Shi, Department of Orthopaedics, Mianzhu Peopl’s Hospital, 618200, China

**Keywords:** Mean platelet volume, Neutrophil-to-lymphocyte ratio, Pulmonary infection, Platelet-to-lymphocyte ratio, Red blood cell distribution width

## Abstract

**Objective:**

To investigate the predictive effects of neutrophil-to-lymphocyte ratio (NLR), platelet-to-lymphocyte ratio (PLR), red blood cell distribution width (RDW), and mean platelet volume (MPV) on the severity of neonatal pulmonary infections.

**Methodology:**

This is a retrospective design. Ninety childrens with pulmonary infection who received treatment in Mianzhu People’s Hospital from March 2023 to September 2024 were randomly selected as the subjects, and were divided into mild, moderate, and severe groups according to the different degrees of pulmonary infection, while 50 healthy newborns were selected as controls. The levels of NLR, PLR, RDW, and MPV were measured, and correlation analyses were conducted.

**Results:**

NLR, PLR and RDW levels were the highest in the severe group, and the MPV level was the lowest (P < 0.05). Serum NLR, PLR, and RDW levels all showed a significant positive correlation with the neonatal critical illness score (NCIS) and oxygenation index (OI) (P < 0.05) and a significant negative correlation with the respiratory index (RI) (P < 0.05); the serum MPV level showed a significant negative correlation with the NCIS and OI (P < 0.05) and a significant positive correlation with the RI (P < 0.05). The area under the curve was 0.710 for NLR, 0.713 for PLR, 0.714 for RDW, and 0.725 for MPV.

**Conclusion:**

NLR, PLR, RDW, and MPV can evaluate the condition of neonatal pulmonary infection and provide a reference basis for clinicians to understand the severity of the condition and rational treatment of children, which has high clinical application value.

## INTRODUCTION

Neonatal pulmonary infection is a relatively common disease in clinical pediatrics, mainly caused by bacterial and viral invasions induced by incomplete immune system development, weak body defenses, and poor organ function after separation from the mother.^1^,^2^ When neonatal pulmonary infection occurs, the quality of the child’s breathing is severely affected, which may lead to respiratory distress, hypoxemia, and even respiratory and cardiac failure. If effective and reasonable treatment is not taken in time, the life of the child may be directly endangered. However, it is worth noting that the early clinical symptoms of this disease are atypical, and it is characterized by rapid onset, high treatment difficulty, and high mortality rate. Previously, neonatal pulmonary infection was diagnosed by chest X-ray or computed tomography and specific laboratory indicators; however, the compliance of neonates and their families with imaging is low, and excessive exposure to ionizing radiation has a high risk of carcinogenesis and immune system damage in neonates.^3^,^4^ Therefore, there is an urgent need to find a safe examination method that is suitable for neonates to prevent further deterioration and ensure the safety of the child.

Blood examination is a routine clinical examination, and complete blood count is easy to obtain. In recent years, some optimized indicators based on blood route indices, such as neutrophil-to-lymphocyte ratio (NLR), platelet-to-lymphocyte ratio (PLR), red blood cell distribution width (RDW), and mean platelet volume (MPV), have been considered to have significant predictive effects in the prognosis of various diseases, including cardiovascular and cerebrovascular diseases and malignancies.^5^-^7^ Numerous studies in China and abroad have confirmed that the above indicators have predictive value in various diseases.^8^-^11^

However, there are few studies on NLR, PLR, MPV, and RDW in assessing the severity of neonatal pulmonary infections. Therefore, this study analyzed the correlation and predictive value of NLR, PLR, RDW, and MPV with the severity of neonatal pulmonary infections in order to provide more theoretical basis and experimental basis for the clinical diagnosis and treatment of neonates.

Our objective was to investigate the predictive effects of neutrophil-to-lymphocyte ratio (NLR), platelet-to-lymphocyte ratio (PLR), red blood cell distribution width (RDW), and mean platelet volume (MPV) on the severity of neonatal pulmonary infections.

## METHODOLOGY

This study is a retrospective design. Ninety children’s with pulmonary infection who received treatment in Mianzhu People’s Hospital from March 2023 to September 2024 were randomly selected as the study subjects, and they were divided into mild, moderate, and severe groups according to the different severity degrees of pulmonary infection, with 30 cases in each group. In addition, 50 healthy newborns born in our hospital during the same period were selected as the control group.

*Ethical Approval:* This study was reviewed and approved by the Ethics Committee of the hospital (MZPH20241118014; dated November 18, 2024).

### Inclusion criteria:

- Delivery in our hospital.
- Diagnosis of pulmonary infection by clinical examination according to the relevant diagnostic criteria of Practical Neonatology.
- Birth age less than 28 days.
- Symptoms such as grunting, cyanosis or refractory apnea of different degrees.
- Inspiration frequency > 60 times/min.
- Infiltrative shadows of different sizes in the lungs on radiography.
- Pathogenic bacteria after culture of lower respiratory tract secretions.
- Parents of the infant were informed and signed the informed consent form.

### Exclusion criteria:

- Chromosomal abnormalities.
- Incomplete clinical data.
- Congenital respiratory malformations.
- Congenital heart disease.
- Congenital metabolic and genetic diseases.

Criteria for determining the severity of pulmonary infection are as follows. Mild: good general condition, no high-risk factors, stable vital signs, and no obvious abnormalities in organ function; moderate: obvious clinical symptoms, mechanical ventilation ≤ 4 days; severe: impaired consciousness, respiratory rate > 30 times/min, arterial partial pressure of oxygen (PaO_2_) < 60 mmHg, PaO_2_/fraction of inspiration O_2_ (FiO_2_) < 300 mmHg, requiring mechanical ventilation or even admission to the intensive care unit (ICU).

### Research methodology:

Using a vacuum blood collection tube containing ethylenediaminetetraacetic acid, 3 ml. of fasting venous blood was collected from infant patients that did not undergo antibiotic therapy and healthy neonates on the morning of the second day of enrollment. Blood analysis was performed using a Beckman AU5800 automated biochemical analyzer. Results of routine blood tests were recorded to obtain serum indicators such as NLR, RDW, PLR, and MPV.

### Observed indicators:

Pulmonary function indicators, including oxygenation index (OI), respiratory index (RI)), and neonatal critical illness score (NCIS), were recorded for newborns in the mild, moderate, and severe groups.^12^

Serum NLR, PLR, RDW, and MPV levels of newborns in the mild, moderate, severe, and control groups were recorded.

Pearson correlation analysis was performed on the correlation between serum NLR, PLR, RDW, and MPV levels in newborns with pulmonary infections and NCIS, OI, and RI. (4) Receiver operator characteristic (ROC) curves were plotted to clarify the predictive value of NLR, PLR, RDW, and MPV levels for neonatal pulmonary infection.

### Statistical analysis:

SPSS26.0 statistical software was used for data analysis. Measurement data conforming to normal distribution were expressed as mean ± standard deviation (X̄±*s*). One-way analysis of variance was used to compare measurement data among multiple groups. The SNK-q test was used for pairwise comparisons between groups. Pearson correlation analysis was used to analyze the correlation between variables. ROC curves were plotted to analyze the predictive value of NLR, PLR, RDW, and MPV levels in neonatal pulmonary infection. The difference was statistically significant at P < 0.05.

The calculation formula for the sample size is:


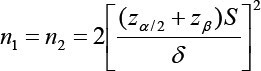


where *n*_1_ and *n*_2_ are the content needed by two samples, *S* is the estimated value of two population standard deviations, and *δ* is the difference between two means, *δ* = *μ*_1_ - *μ*_2_.

## RESULTS

There were no statistically significant differences in the general data such as age, weight, and gender of newborns in each group (Table-I). The levels of NLR, PLR, and RDW of newborns in the severe group were higher than those in the moderate, mild, and control groups, and the level of MPV was lower than that in the moderate, mild, and control groups; the differences were statistically significant (P < 0.05). The levels of NLR, PLR, and RDW in the moderate and mild groups were higher than those in the control group, and the level of MPV was lower than that in the control group; the differences were statistically significant (P < 0.05) (Table-II). In the infected groups, the NCIS score and OI decreased significantly and the RI increased significantly (P < 0.05) as the disease condition worsened, as shown in Table-III.

**Table-I:** Comparison of general data between different groups of newborns.

Group	n	Embryonic age (week)	Age in days (d)	Gender (male/female)	Body weight (g)
Severe group	30	30.52±53.57	15.32±2.14	(18/12)	1951.52±123.46
Moderate group	30	31.02±6.06	16.03±3.51	(17/13)	1980.76±150.05
Mild group	30	30.48±53.62	15.57±2.36	(16/14)	1974.48±131.57
Control group	50	30.58±52.71	15.93±2.41	(27/23)	1954.29±125.38
F		0.984	1.628	0.169	1.514
P		> 0.05	> 0.05	> 0.05	> 0.05

**Table-II:** Comparison of serum NLR, PLR, RDW, and MPV levels in newborns in different groups (***Χ̅**±S*).

Group	n	NLR	PLR	RDW (%)	MPV
Severe group	30	11.63±5.70^abc^	261.30±155.65^abc^	16.58±3.53^abc^	9.06±1.05^abc^
Moderate group	30	7.46±3.28^ab^	213.72±86.47^ab^	15.46±2.79^ab^	9.31±0.98^ab^
Mild group	30	3.04±0.89^a^	162.38±57.52^a^	14.50±2.63^a^	9.52±0.76^a^
Control group	50	1.85±0.88	120.83±50.72	13.08±0.47	9.84±0.85
F		13.115	12.472	13.761	9.178
P		< 0.05	< 0.05	< 0.05	< 0.05

***Note:*** a: compared with the control group, P < 0.05; b: compared with the mild group, P < 0.05; c: compared with the moderate group, P < 0.05.

**Table-III:** Comparison of pulmonary function indicators and NCIS in newborns with different levels of infection (***Χ̅**±S*).

Group	n	NCIS	OI (mm Hg)	RI
Severe group	30	62.44±2.93^bc^	187.57±8.48^bc^	2.36±0.32^bc^
Moderate group	30	80.72±2.94^b^	226.39±8.50^b^	2.12±0.35^b^
Mild group	30	93.27±2.96	268.36±8.52	1.87±0.33
F		14.325	17.937	12.912
P		< 0.001	< 0.001	< 0.001

***Note:*** b: compared with the mild group, P < 0.05; c: compared with the moderate group, P < 0.05.

Pearson correlation analysis showed that serum NLR, PLR, and RDW levels of newborns with pulmonary infections were significantly and positively correlated with NCIS and OI (P < 0.05), and negatively correlated with RI (P < 0.05); the serum MPV level was significantly and negatively correlated with NCIS and OI (P < 0.05), and positively correlated with RI (P < 0.05) (Table-IV).

**Table-IV:** Correlation analysis of NLR, PLR, RDW and MPV levels with NCIS, OI and RI.

Indicator	NCIS		OI		RI	
	r value	P value	r value	P value	r value	P value
NLR	0.843	0.001	0.674	0.006	-0.852	0.001
PLR	0.769	0.002	0.832	0.001	-0.857	0.001
RDW	0.784	0.001	0.688	0.004	-0.754	0.002
MPV	-0.825	0.001	-0.734	0.002	0.863	0.001

**Fig.1:**
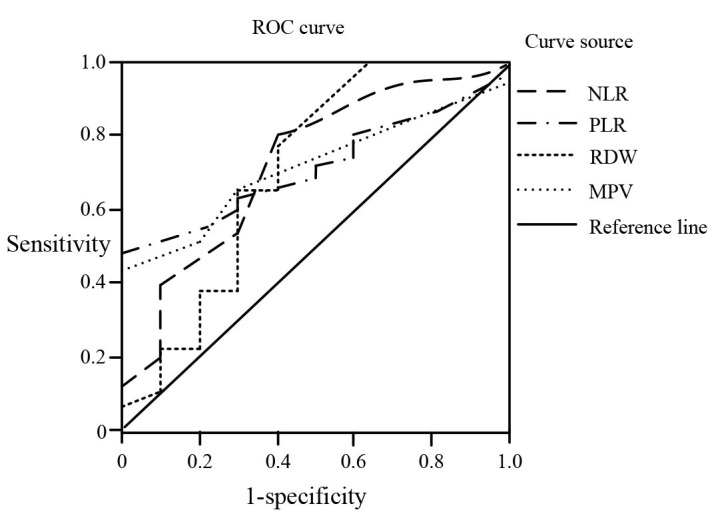
Curves of NLR, PLR, RDW, and MPV for the diagnosis of pulmonary infections.

The levels of NLR, PLR, RDW, and MPV of the newborns were included in the ROC curve analysis. The area under the ROC curve of NLR was 0.710 (95% CI: 0.560-0.863, P < 0.05). The area under the ROC curve of PLR was 0.713 (95% CI: 0.493-0.930, P < 0.05). The area under the ROC curve of RDW was 0.714 (95% CI: 0.562-0.864, P < 0.05). The area under the ROC curve of MPV was 0.725 (95% CI: 0.538-0.910, P < 0.05) (Fig.1).

## DISCUSSION

In this study, four indicators, namely NLR, PLR, RDW and MPV, were used to evaluate and predict neonatal pulmonary infections. The results showed that the levels of NLR, PLR, and RDW in the infected groups were higher than those in the control group, and the level of MPV was lower than that in the control group. It indicated that the levels of NLR, PLR, RDW, and MPV might reflect the severity of pulmonary infection. The reason for this result is as follows. The inflammatory response in the blood vessels during pulmonary infection may lead to vascular dysfunction and hypoxia, resulting in atherosclerosis and airflow obstruction in the arteries, which causes various changes in the ratio of platelets to lymphocytes and also increases RDW. In addition, because infection and hypoxia cause damage to pulmonary vascular endothelial cells and exposure of subendothelial collagen, platelets are activated, leading to changes in MPV. A study by Kurt RK et al. suggested that RDW was significantly higher in pregnant women with pre-eclampsia.^13^ They also found that RDW was associated with the severity of the disease. The results of the study by Cao et al. suggested that the MPV level in patients with acute exacerbations of chronic obstructive pulmonary disease (AECOPD) decreased as the patients’ disease worsened.^14^ Şahin et al. found that NLR, PLR, and RDW values, as inflammatory markers in patients with AECOPD, can be used to predict the deterioration of COPD and the severity of future exacerbations.^15^ The above research results all indicate that the indicators of NLR, PLR, RDW, and MPV have the ability to predict the severity of the disease, which is similar to the results of this study. However, the research subjects of the above studies are not targeted at newborns with pulmonary infections. The predictive value of NLR, PLR, RDW, and MPV in neonatal pulmonary infections has been rarely reported in existing Chinese and foreign studies.^16^-^19^ The results of this study show that these indicators can also indicate the severity of neonatal pulmonary infections, providing effective evidence for clinicians to formulate treatment plans for children.

In addition, this study was the first to analyze the correlation between NLR, PLR, RDW, and MPV values and NCIS, OI, and RI. OI is a common clinical indicator of oxygenation in children with infectious pneumonia; a lower OI indicates severer hypoxia and lung injury.^20^ RI is not affected by the mode of oxygen delivery and volume fraction, so it is more accurate in assessing the actual oxygen and status, and is a common indicator for clinical assessment of pulmonary ventilation and gas exchange.^21^ The results of this study showed that serum NLR, PLR, and RDW levels in newborns with pulmonary infections were significantly and positively correlated with NCIS and OI (P < 0.05) and significantly and negatively correlated with RI (P < 0.05); the serum MPV level was significantly and negatively correlated with NCIS and OI (P < 0.05) and significantly and positively correlated with RI (P < 0.05). It further demonstrated that NLR, PLR, RDW, and MPV levels can accurately assess the severity of pulmonary infections in newborns, this is also the innovation of this study, which can provide new ideas and references for the clinical diagnosis and treatment of children with pulmonary infection.

### Limitations:

It is a single-center study with a single source of samples and a relatively small sample size. It is unable to rule out the influences of aspects such as region, environment, and race, resulting in certain limitations in the epidemiological characteristics. The results of this study may be subject to errors due to the relatively small sample size and some limitations associated with retrospective studies.

## CONCLUSION

The levels of NLR, PLR, RDW, and MPV can accurately reflect the severity of neonatal pulmonary infection and have high predictive value. The detection of these four indicators can provide a reference for clinicians to accurately assess the condition of newborns and develop the best subsequent treatment plan, which is worthy of clinical promotion and application.

### Recommendations

In future research, attention will be paid to expanding the sample size and designing experimental methods to minimize research errors as much as possible.

### Authors’ Contribution:

**JD:** Study design, data collection and analysis.

**JD & QLS:** Manuscript preparation, drafting and revising.

**JD & QLS:** Review and final approval of manuscript.

All authors have read and approved the final version of the manuscript and they are also accountable for the integrity of the study.
